# An Ishmaelite

**Published:** 1888-06-30

**Authors:** Leslie Keith

**Affiliations:** Author of "The Chilcotes," "Uncle Bob's Niece," "East and West," etc.


### AN ISHMAELITE.

BY LESLIE KEITH,

Author of "The Chilcotes," "Uncle Bob's Niece," "East and West," etc.

(*Continued from p. 202.*)

### CHAPTER XII.—EFFECTS OF A LIBRARY.

A STUDENT of character could not well select a better place than a circulating library for his observations. A man's choice of books betrays him perhaps even more surely than his choice of friends: one's friends being, indeed, rarely the result of a deliberate choice at all. To be sure, there are people who never know what they want to read, and who need to have their minds made up for them, but there are also many who at once find their spiritual kin waiting to reveal themselves in that valhalla of dead and living heroes—a library.

Both classes were present among the clients who patronized the new venture in Gillespie Street. The library very soon became popular, and it was apparent that it supplied what advertisers call "a felt want." Curiosity, however, brought a great many people to begin with, who did not know that they had a want till it was pointed out to them. It was certainly curiosity that induced the Miss Greens to head the crowd; they came so early, indeed, that the assistant was busy putting some last finishing touches on a high shelf, and did not hear their entrance. He was presently aware of an unusual rustling and giggling, however, and came down the steps.

Miss Maria looked at him, and an expression of unmistakable disappointment crossed her face. She decided rapidly that a new hat, worn for his subjection, was quite thrown away on this grey, grim man.

"Can I do anything for you?" asked Fred, remembering his duties.

"We've come to look at the Libe'ry," said Miss Maria, with a toss of the new hat. "My! what a lot of books. I suppose you know the names of 'em all?"

"I think so."

"Tell us some of 'em. What's that lot?" She pointed with her parasol to a brown-papered row neatly lettered in Delia's round hand on the back.

"That is history. Freeman, Macaulay, Green—"

Miss Bell giggled. "Green—that's our name," she said, "but it wasn't any of our family that wrote it."

"Oh, dry, stupid stuff," said Miss Maria, dismissing the historians with contempt. "Have you any poetry? I dote on poetry."

"Yes, the poets are pretty well represented," said Fred, beginning to take a faint amusement over his task. "This bookcase is nearly filled with them. Shakespeare, Milton, Scott, Byron, Browning." He ran his hand down the shelves.

"Lor!" cried Miss Maria, opening a pair of light-blue eyes, "I never knew there was so much poetry wrote before. Do you know Nimrod, who writes the verses in the *Morning Star?* They're what I call sweet. I should love to know him; I'm sure it's *him*, from the way he writes about ladies," she simpered.

Eastgate modestly confessed that he was not acquainted with Nimrod's muse, and thereby fell many degrees in her esteem. Miss Bell was meanwhile investigating the inner room.

"I shouldn't mind taking a novel, if you've got a good one," she said, in a languid voice.

"What kind do you prefer," asked the librarian, joining her.

"Oh, I ain't pertickler, we take the *Fam'ly 'erald* and the *Story Teller*. I like something spicy, none of your dull, preaching sort."

When Barbara came down, Fred broke through his usual reticence to tell her of the visit.

"What did you give her?" asked Barbara, with interest. But when he named his choice, she shook her head, and her lips curled into a little smile.

"She won't thank you," she said; "it's the battle, murder, and sudden death order of literature she has been used to."

"Then isn't it time her taste was corrected?" he began impetuously, but he suddenly pulled himself up. "I shall learn in time, I—I am new to the business. I hope you will have patience with me," he said submissively.

"Oh," said Barbara, "you know far, far more than I, but perhaps for that very reason you can't guess how very little we know—we who live here; and if you could see some of the homes—not the very poorest either, you would understand how it is that the boys and girls want to be taken out of themselves into a livelier world, where they can imagine themselves rich and grand and beautiful for a little while."

"But it is a false view of life."

"Yes," she said, "I feel that; I should be sorry to think that the stories our boys and girls like best are true. I never saw a duke or a duchess," said the girl merrily, "or even a baronet's wife, but if I wanted to imagine one I shouldn't dress up Mrs. Green in silks and satins and put her in a drawing-room; but I've made up my mind not to be disheartened by the books we are asked to lend at first.

That's why I asked father to let us have all kinds except the bad. We mustn't frighten them, but if we start with the trashier sort, we may by and by get the better ones smuggled in, like the pills in the jam."

Barbara had never been so cheerful and gay, and as Fred looked at her he wondered he could ever have thought her plain, with those intelligent grey eyes and that broad brow. Barbara was indeed in harmony with her surroundings, she was by nature a lover of books, and had, by a happy instinct, pastured only on the best. She and Delia had never gone to school, they had been educated privately and well, by a lady whom Proudie chose with an anxious care, placing, in common with most of his countrymen, a high value on book learning, he had had the good sense to set his face against those perfunctory "tinkling and smearing" accomplishments over which so much time is misspent; and the result justified his decision.

Fred soon learnt that Barbara's judgment was quite correct; there were readers of all sorts, it is true—intelligent working men and young clerks, anxious to improve their opportunities, who tackled the strong meat of literature, and knew how to value it; but the crowds of young folk who are, after all, the main supporters of a circulating library, showed no very elevated appreciation of the fare provided for them.

The girls were worse than the boys. The latter were readily enough satisfied with thrilling tales of adventure and peril that fired their young blood and sent their fancy roving; but the girls—they stuck to their languishing fine ladies, and their impossible lords, poor little women, and would have "high life" from the back kitchen point of view, or none at all. The dust gathered upon many a worthy author pining in oblivion on those upper shelves, while others were flatteringly thumbed and dog-eared and wept over, perhaps, by sentimental maidens, whose foolish little hearts were filled with dreams of a conquering Lovelace about to rise on their horizon.

When one or two of those special favourites fell into Miss Betsy's hands to receive new garments of brown paper, she put on her spectacles and examined the contents, curious to discover the secret of their evident popularity. This stern moralist had read scarce more than a page or two before her indignation flamed out.

"Barbara Proudie," she said, looking at her elder niece, who sat on the opposite side of the table engaged on the same business, "I wonder you're not ashamed to sit there and look an honest single woman in the face. If your education has done no more for you than to teach you to read trash and nonsense like this, and pass it on to the poor deluded lassies, you might better have been a heathen savage."

"Oh, auntie," said Barbara, blushing under this accusation, "What have you got hold of? Oh, that? Yes, it is pretty foolish, I admit. It is about the worst. I think we might withdraw it from circulation now."

"Foolish!" cried the wrathful Miss Betsy, "it's fair pernee-cious." Miss Betsy's indignation was not easily appeased. She lit a candle and marched down at once upon the astonished librarian, who generally spent his evenings in solitude among the books. She put him through an awful catechism, she stung him with many a home thrust. If the grim maiden lady had had her way, there would have been scarce a love-story or a poem that was safe from her auto-da-fé. The offending volume was solemnly burned and the wondering Delia, who had been called to witness this ceremony, was warned off those shelves where the writers of fiction congregated.

"We must let them have love stories," said Barbara, half ruefully, half comically; "and I was getting them led along so nicely to the better kind of ones. Do the girls in the West-end read nothing but history and science and tracts, Mr. Eastgate?"

"I don't know," said Fred, reddening violently and hanging his head.

Barbara saw that she had somehow pained him, and she could have bitten her tongue out. She would not willingly have hurt a fly. It was Barbara's rare tact that was the first quality Fred took notice of in his companion. He wondered at it in a strange, dull way. It was the best sort of tact—the tact borne of insight and sympathy, and it taught her not only to know instinctively what each applicant wanted, but how to treat each too. There were some whose weekly score she allowed to run on unpaid, and others whose fees she remitted altogether, while there were a few with whom she was very stern and relentless.

"They mustn't come here again," she said, "It isn't the books they come for."

Fred wondered what she meant. He had not lived in Bermondsey all his life: of the surrounding homes he knew nothing—of their tragedies, their joys, their pleasures, their temptations. Barbara was wiser than he in this order of knowledge, and though her natural and noble pity made it her impulse to bury in secret the lowest fatalities of her neighbours, her severity towards certain lapses was scarcely less keen than her pity. Fred wondered sometimes at the girl's sad, stern face, and wondered, too, at the love and kindness that could beam out of it. He began to watch the people as they came, and to try to look at them through her eyes, and gradually the habit of getting interested in others grew and slowly gathered strength. To find the world still good, though he had parted with his own birthright in it, to lose something of his self-murmuring bitterness in a widening sympathy for other strugglers—surely this was no bad lesson for the poor prodigal to learn in the humble refuge where he had hidden himself.

The library gave him scope enough for those new studies, for under a sordid general monotony of vulgarity and poverty there lay hid an immense variety of history and character. Barbara sketched for him some of those little biographies.

"Be good to this poor lady," she would whisper. "Yes, she is a lady; she is not like us: seek out a nice book for her."

Barbara herself had a bright word for the ancient damsel, who wore a sad air of battered and war-worn gentility in her faded dress and shrunken features.

"Dear Miss Crossfield," she said, "you have come to save us from reproach. Here is *Middlemarch* sulking in a corner, because nobody wants to be introduced to it; and such a fine, new, clean copy!"

"Ah, my dear,' said the little lady, nodding a mysteriously-ornamented bonnet, "I have some sense of literature, I hope, and some notion of an author's quality. I will not disgrace the introduction."

"Do rest a minute," said Barbara, as she secured the book and inserted the borrower's name in the ledger, "Delia is going to bring us some tea. Living among learning makes one very thirsty, I find; and it is hot, isn't it?"

"My dear, I can't wait for Delia and the tea, and as for the heat, you have given me something to make me forget it." She went away with a salutation that included Eastgate, and an undaunted cheerfulness animating her forlorn finery, that seemed to move Barbara strangely.

"Oh, it is a shame! a shame!" she said vehemently.

Fred looked at her wonderingly, and she arrested herself with a laugh, but there were tears in her eyes."

"It is fate I am railing at, she said, "fate that has conquered that poor little lady. Some day I will tell you her history. Did you notice her dress?"

"No," said Fred, stupidly, "I'm afraid not."

"Oh, you are a man; it wouldn't strike you. Well, never mind, you noticed her face, I dare say? She hasn't enough to eat, not nearly enough; and," said the girl with a sort of vehemence, "I never can get her to take anything here. I've tried it at the cost of all sorts of prevarications; I'm always hungry when she comes, and I think she detects me!" she laughed rather unsteadily. "It is dreadful to be so proud, it is a great price to pay for being a lady."

He looked at her with an absent disregard of all except her concluding words.

"Why do you always talk as—as if you were not one?" he asked.

"Because," she said, with a shade of coldness, "I am not one." Then, with a sudden change back to her merrier mood, she added—

"It is on my conscience to send for that tea that I said was coming. Will you help me to drink it, Mr. Eastgate?"

"No," he said abruptly, "don't ask me, I cannot."

He went quickly into the further part of the shop, and busied himself fiercely there, though he knew that her surprised looks followed him.

"I will not let her innocent kindness lead her into doing what she may one day repent," he said. "If she knew who and what I am, would she tolerate my presence even as her servant?" The thought that one day Barbara might know, lay like a dark shadow on his spirit. If Barbara should ever know, would she judge and condemn him in her young and stern severity, or would there dawn upon her face for him that angel-like look of pity he had seen it wear for other sinners who had fallen by the way?

*(To be continued.)*

